# Comparison of the tumor immune microenvironment and checkpoint blockade biomarkers between stage III and IV non-small cell lung cancer

**DOI:** 10.1007/s00262-022-03252-y

**Published:** 2022-07-26

**Authors:** Yinjie Gao, Michelle M. Stein, Matthew Kase, Amy L. Cummings, Ramit Bharanikumar, Denise Lau, Edward B. Garon, Sandip P. Patel

**Affiliations:** 1 Tempus Labs, Chicago, IL 60654 USA; 2 UCLA School of Medicine, Los Angeles, CA 90095 USA; 3 UC San Diego Health, La Jolla, San Diego, CA 92093 USA

**Keywords:** Immunotherapy, Checkpoint blockade, PD-L1, Transcriptomics, Non-small cell lung cancer, Tumor immune microenvironment

## Abstract

**Background:**

Adjuvant immune checkpoint blockade (ICB) following chemoradiotherapy and adding ICB to chemotherapy have been key advances for stages III-IV non-small cell lung cancer (NSCLC) treatment. However, known biomarkers like PD-L1 are not consistently indicative of ICB response. Other markers within the tumor immune microenvironment (TIME) may better reflect ICB response and/or resistance mechanisms, but an understanding of how TIMEs differ between stage III and IV NSCLC has not been explored.

**Methods:**

Real-world data from unresectable, stage III-IV, non-squamous, pretreatment NSCLCs (stage III *n* = 106, stage IV *n* = 285) were retrospectively analyzed. PD-L1 immunohistochemistry (IHC) was compared to *CD274* gene expression. Then, differential gene expression levels, pathway enrichment, and immune infiltrate between stages were calculated from whole-transcriptome RNA-seq. Analyses were stratified by *EGFR* status.

**Results:**

PD-L1 IHC and *CD274* expression in tumor cells were highly correlated (*n* = 295, *P* < 2.2e-16, *⍴* = 0.74). *CTLA4* expression was significantly increased in stage III tumors (*P* = 1.32e-04), while no differences were observed for other ICB-related genes. Metabolic pathway activity was significantly enriched in stage IV tumors (*P* = 0.004), whereas several immune-related KEGG pathways were enriched in stage III. Stage IV tumors had significantly increased macrophage infiltration (*P* = 0.0214), and stage III tumors had a significantly higher proportion of CD4 + T cells (*P* = 0.017). CD4 + T cells were also relatively more abundant in *EGFR*-mutant tumors vs. wild-type (*P* = 0.0081).

**Conclusion:**

Directly comparing the TIMEs of stage III and IV NSCLC, these results carry implications for further studies of ICB response in non-resectable stage III NSCLC and guide further research of prognostic biomarkers and therapeutic targets.

**Supplementary Information:**

The online version contains supplementary material available at 10.1007/s00262-022-03252-y.

## Introduction

The addition of adjuvant or consolidative immune checkpoint blockade (ICB) following concurrent chemoradiation was the key practice-changing development for locally advanced non-small cell lung cancer (NSCLC) in the last decade. Recently published 5-year survival results from the PACIFIC study demonstrate patients who initiate ICB early carry a significant risk reduction for 5-year overall survival [1]. Additionally, the IMpower010 and CheckMate 816 trials have shown survival benefit in even earlier-stage patients, where ICB plus chemotherapy was favorable compared to chemotherapy alone in patients with stage IB-IIIA NSCLC [2, 3]. These data suggest an approach leveraging both ICB and chemotherapy in the first line may be more efficacious. Importantly, this combination strategy has significantly improved survival rates of patients with stage III or IV NSCLC. However, across both stages, we do not have a strong understanding of which tumor immune microenvironment (TIME) signatures are associated with response or resistance to ICBs. Furthermore, although ICB is a promising avenue for improving the treatment of stage III NSCLC, some clinical trials have shown negative results for primary endpoints and/or reported higher than expected rates of grade 3/4 adverse events, such as pneumonitis [4–7].

Several TIME mechanisms may contribute to response, resistance, or immune-related toxicities following ICB administration and help identify patient populations likely to experience durable response. The current standard in assessing eligibility for ICB treatment in NSCLC is levels of PD-L1 protein in the tumor, as commonly prescribed ICBs directly target PD-L1 or its receptor PD-1. While PD-L1 scores from immunohistochemistry (IHC) may enrich prediction of response to ICB monotherapy or combination therapy in advanced NSCLC, some evidence suggests responders are not limited to those with high PD-L1 expression [8]. For instance, a recent meta-analysis of 7,617 patients from ICB clinical trials concluded that a subset of patients defined as PD-L1 negative still benefit from ICBs [9]. Other FDA-approved biomarkers such as tumor mutational burden (TMB) and microsatellite instability (MSI) have shown only modest predictive ability, illustrating the clear, ongoing need for better biomarkers of ICB response in NSCLC [10]. TIME-related markers and genetic signatures increasingly have been associated with response to ICB and may offer more comprehensive biomarkers to account for the complexity of ICB treatment [8, 10–13].

An understanding of how TIMEs differ between locally advanced and metastatic NSCLC has not been thoroughly explored, and knowledge of those differences will be crucial to optimizing therapeutic efficacy. Furthermore, studies including large-scale, real-world data are needed to assess the molecular landscape of patients who may be ineligible for controlled trials due to restrictive inclusion criteria [14].

Combining RNA-seq, DNA-seq, IHC, and real-world clinical data from the Tempus Database, we retrospectively analyzed a cohort of pretreatment tumors from patients with unresectable stage III and IV non-squamous NSCLC. We provide a comparison of the TIME in stage III-IV NSCLC by using whole-transcriptome RNA-seq to measure relative gene expression levels, pathway enrichment, and immune infiltrate proportions. To our knowledge, this direct comparison of RNA data between locally advanced and metastatic NSCLC has yet to be described in the literature. In addition, we presented an evaluation of PD-L1 (*CD274*) levels from both IHC and RNA-seq data among the cohort. Together, this study supplies a comprehensive characterization of the immune landscape of unresectable stage III and stage IV NSCLC tumors and may guide future research of ICB biomarkers and novel drug discovery.

## Results

### Cohort overview

**Fig. 1:**
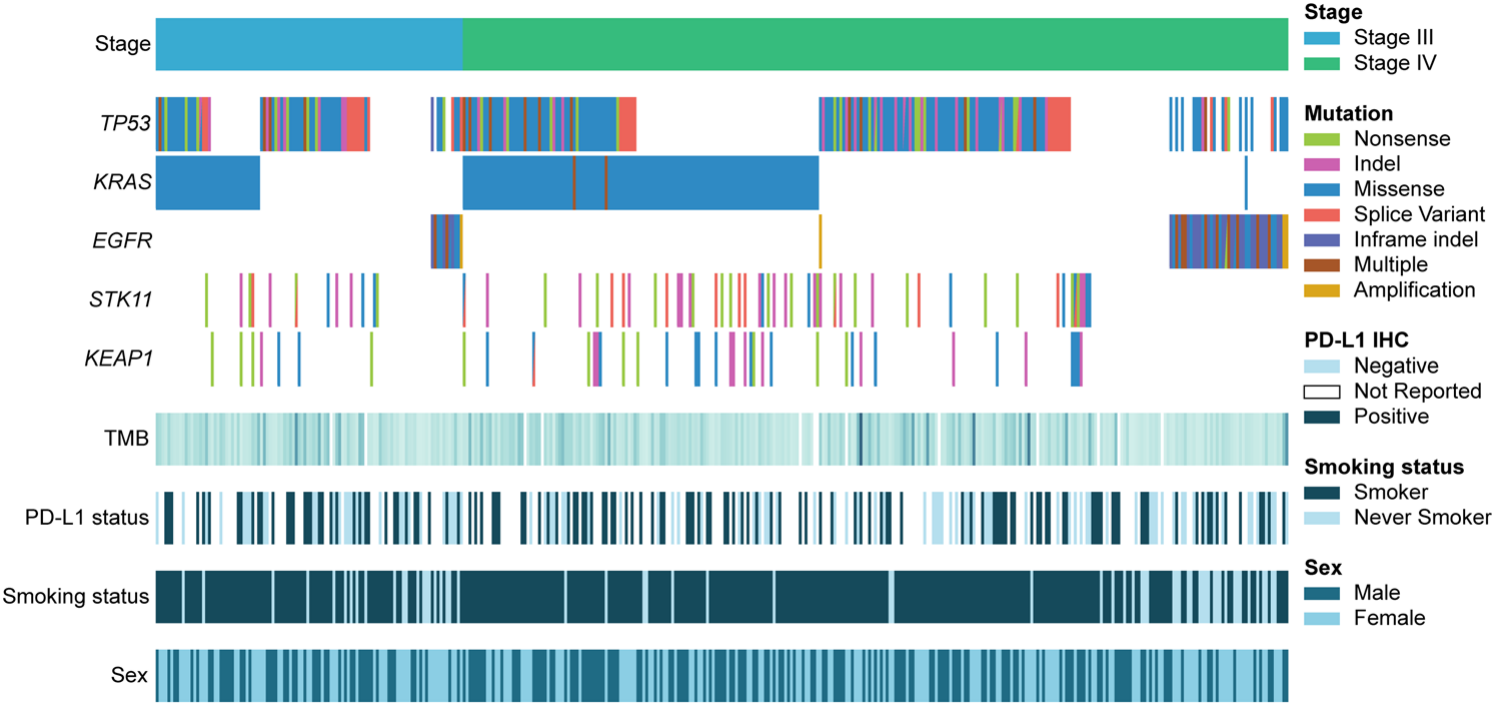
Clinical and genomic characteristics of the NSCLC cohort by tumor stage (stage III *n* = 106, stage IV *n* = 285). Each column represents a tumor in the above CoMut plot. Tumors are ordered by stage and by mutation clusters generated by hierarchical clustering (cluster assignments not shown) for clearer visualization of driver mutation patterns. Mutations in *TP53*, *KRAS*, *EGFR*, *STK11*, and *KEAP1* are shown for each tumor sample. Darker colors represent increasing tumor mutational burden (TMB) in the TMB row. Patients were considered positive for PD-L1 if percent tumor cell staining (tumor proportion score) was > 1%, determined from IHC. Former and current smokers were considered smokers. Smoking status was imputed for approximately 10% of patients (see Methods)

A representative sample of 400 de-identified health records from patients with non-squamous, unresectable stage III-IV NSCLC was selected from the Tempus Database. The cohort was restricted to pretreatment tumors to reduce medication-associated transcriptional variation and only included non-squamous histology to limit molecular heterogeneity within stages. After applying inclusion criteria based on histology and biopsy procedure, where biopsies from the lung and/or airway were considered as primary and included (see Methods: *Cohort Selection*), the final stage III and IV cohorts contained 106 and 285 samples, respectively. Oncogenic driver frequencies, demographics, and clinical characteristics of the cohort are provided in Fig. 1 and Table 1. There were no significant differences in age at biopsy collection, sex, or smoking history between stage III and IV patients. Likewise, the frequencies of common oncogenic driver mutations were similar between stages. *EGFR* mutations were observed in 11% (*n* = 11) of stage III and 15% (*n* = 42) of stage IV tumors, similar to previous large-scale evaluations of real-world NSCLC data [15]. While TMB and tumor purity did not significantly differ between the two stages, *EGFR*-mutant tumors had a significantly lower TMB than *EGFR* wild-type (WT) tumors, reflecting previous reports [15, 16]. To account for this difference and other anticipated *EGFR*-related effects, the cohorts were further divided by *EGFR* status for comparisons between *EGFR-*mutant (stage III *n* = 11 and stage IV *n* = 41) and WT tumors in addition to non-*EGFR*-stratified analyses. Other targetable alterations such as ALK fusions were not subset for analyses as they represented smaller portions of the cohort and could not be adequately powered for statistical comparisons.

**Table 1:** Demographics and clinical characteristics of the NCSLC cohort by tumor stage

Characteristic	Stage III (*n* = 106)	Stage IV (*n* = 285)
Female [*n* (%)]	57 (54%)	138 (48%)
Age [median (IQR)]	69 (62—76)	69 (61—76)
Any smoking history [*n* (%)]	85 (80%)	246 (86%)
*Biopsy tissue source* [*n* (%)]		
Lung	105 (99%)	280 (98%)
Airway	1 (1%)	5 (2%)
*Histology* [*n* (%)]		
Adenocarcinoma	96 (91%)	261 (91%)
Adenosquamous carcinoma	2 (2%)	5 (2%)
Non-small cell carcinoma	8 (7%)	19 (7%)
*Driver Mutations* [*n* (%)]		
*EGFR* pathogenic mutation	11 (10%)	42 (15%)
*STK11* pathogenic mutation	13 (12%)	46 (16%)
*KRAS* pathogenic mutation	36 (34%)	124 (44%)
*KEAP1* pathogenic mutation	8 (7%)	26 (9%)
TMB [median (IQR)]	4.0 (2.1–7.4)	3.8 (2.1–6.8)
Tumor purity [median (IQR)]	42% (31–56%)	41% (31–53%)
^†^HLA-LOH [*n* (%)]	24 (30%)	64 (33%)
^†^HLA class I mutation [*n* (%)]	4 (5%)	20 (10%)

†HLA-LOH and HLA Class I mutation % calculated from total known (stage III *n* = 79 and stage IV *n* = 190)

### PD-L1 IHC and CD274 Expression Levels in the TIME of Stage III-IV NSCLC

**Fig. 2:**
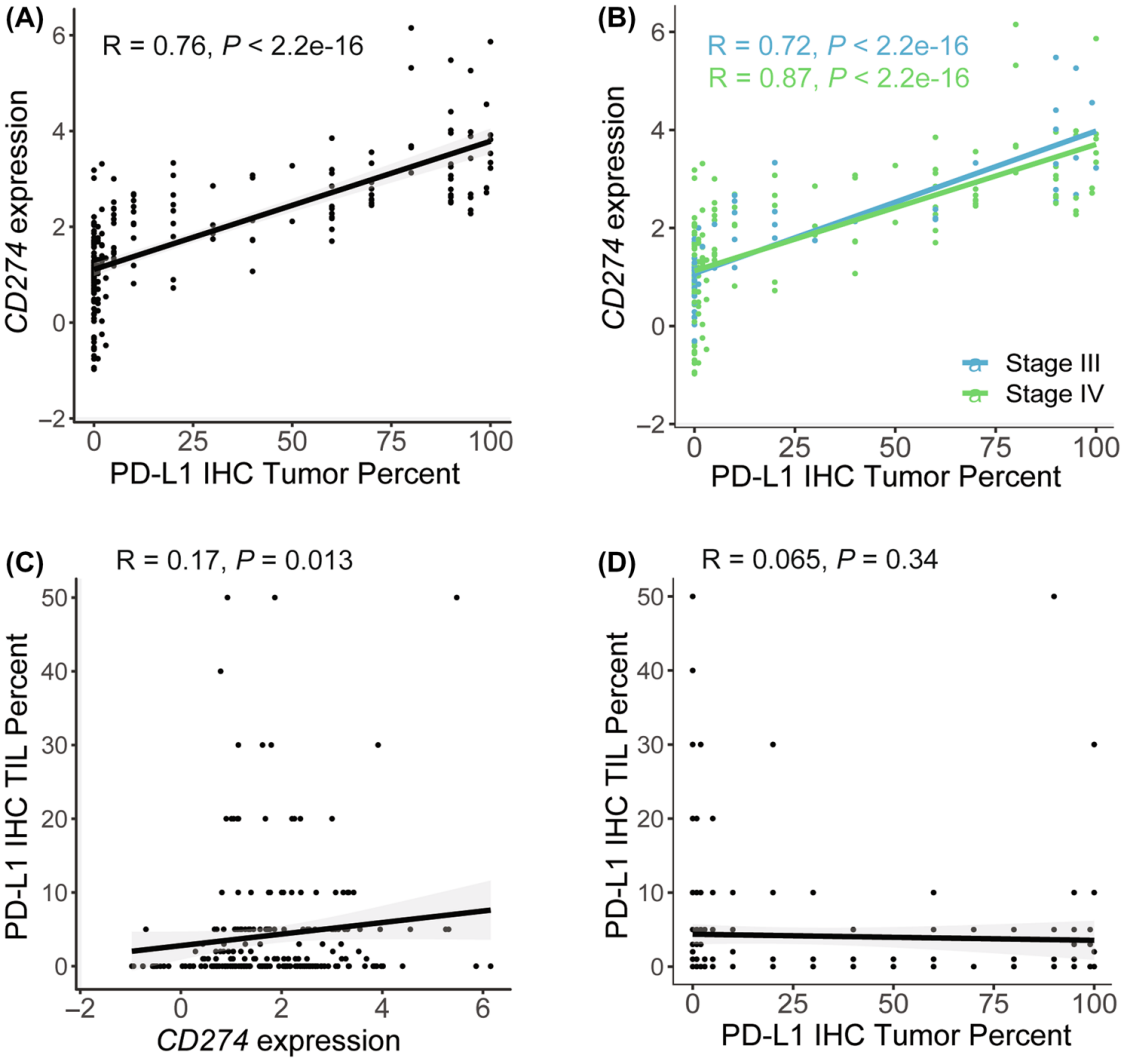
Concordance between PD-L1 from IHC compared to PD-L1 (*CD274*) gene expression levels from RNA-seq. Pearson correlation R and P value noted in top left corner. **a** PD-L1 percent measured on tumor cells (tumor proportion scores) from IHC (*x*-axis) compared to *CD274* gene expression (*n* = 295). **b** PD-L1 concordance between *CD274* gene expression and PD-L1 tumor percent by stage (stage III *n* = 80, stage IV *n* = 215). **c** PD-L1 IHC TIL percent compared to *CD274* (*n* = 222). **d** PD-L1 IHC TIL percent compared to PD-L1 IHC tumor percent (*n* = 222)

PD-L1 IHC staining on tumor cells and/or tumor-infiltrating lymphocytes (TILS) is currently the gold standard eligibility assessment for ICB treatment, but its concordance with expression levels of the gene encoding PD-L1 (*CD274*) is not well established. Thus, we examined the relationship between PD-L1 tumor proportion score (TPS) as measured by the 22C3 IHC assay and *CD274* gene expression from whole-transcriptome RNA-seq of bulk tumor tissue. An overview of PD-L1 IHC scores among stage III and IV tumors by ≥ 1%, ≥ 5%, and ≥ 50% positivity thresholds is presented in Table 2. Across tumors from both stages with available IHC data (*n* = 295), PD-L1 TPS and *CD274* expression in tumor cells were highly correlated (*P* < 2.2e-16, *⍴* = 0.74) (Fig. 2a). This correlation persisted within stage III (*n* = 80, *P* < 2.2e-16, *⍴* = 0.8) and IV (*n* = 215, *P* < 2.2e-16, *⍴* = 0.72) samples (Fig. 2b). In contrast, there was only weak correlation between PD-L1 IHC in TILs and *CD274* expression in whole-tumor samples across the cohort (*n* = 222 with available TIL IHC data, (*P* = 0.008, *⍴* = 0.18), (Fig. 2c). This is likely due to differences in relative immune proportion in the tumor RNA-seq sample, as we observed TIL PD-L1 IHC was modestly correlated with RNA-estimated immune proportion (*P* = 0.0083, *⍴* = 0.18), *CD4* expression (*P* = 0.00083, *⍴* = 0.22) and *CD8* expression (*P* = 6.1e-5, *⍴* = 0.26). Similarly, there was no significant correlation between TIL and tumor cell PD-L1 IHC (*P* = 0.17, *⍴* = 0.9.91) (Fig. 2d).

**Table 2:** Stratification of stage III and IV cohorts by PD-L1 IHC tumor percent cutoffs

PD-L1 IHC Tumor Percent	Number of Stage III Tumors with PD-L1 Score (*n* = 63)	Number of Stage IV Tumors with PD-L1 score (*n* = 155)
Negative (< 1%) [*n* (%)]	28 (44%)	64 (41%)
Low (1–49%) [*n* (%)]	20 (32%)	55 (36%)
High (≥ 50%) [*n* (%)]	15 (24%)	36 (23%)

**Fig. 3:**
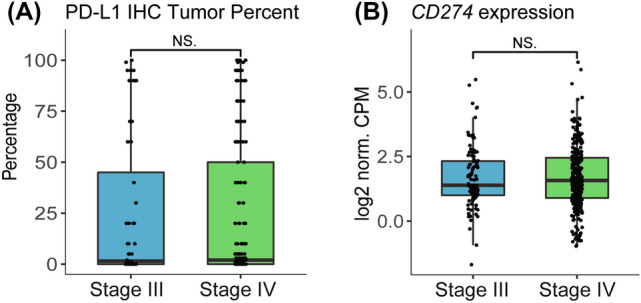
No significant differences in PD-L1 IHC or *CD274* expression were observed between stage III and stage IV tumors. **a** Boxplot of PD-L1 IHC tumor percent by stage. PD-L1 IHC tumor percent was not significantly different between stage III and stage IV NSCLC (stage III *n* = 80, stage IV *n* = 215). **b** Boxplot of PD-L1 (*CD274*) log2 normalized CPM gene expression by stage. *CD274* was not differentially expressed by stage (stage III *n* = 106, stage IV *n* = 285)

After establishing concordance between PD-L1 TPS and *CD274* expression in the combined cohort, we next compared PD-L1 TPS and *CD274* expression by stage. There was no significant difference in PD-L1 TPS (Fig. 3a) or *CD274* expression (Fig. 3b) between stage III and IV tumors. No significant differences by stage in either PD-L1 TPS or TMB were observed after stratifying by *EGFR*, *STK11*, *KEAP1,* or *KRAS* mutation statuses (Supplementary Fig. 1).

### Transcriptome-wide differential expression analysis between stage III and IV NSCLC tumors

**Fig. 4:**
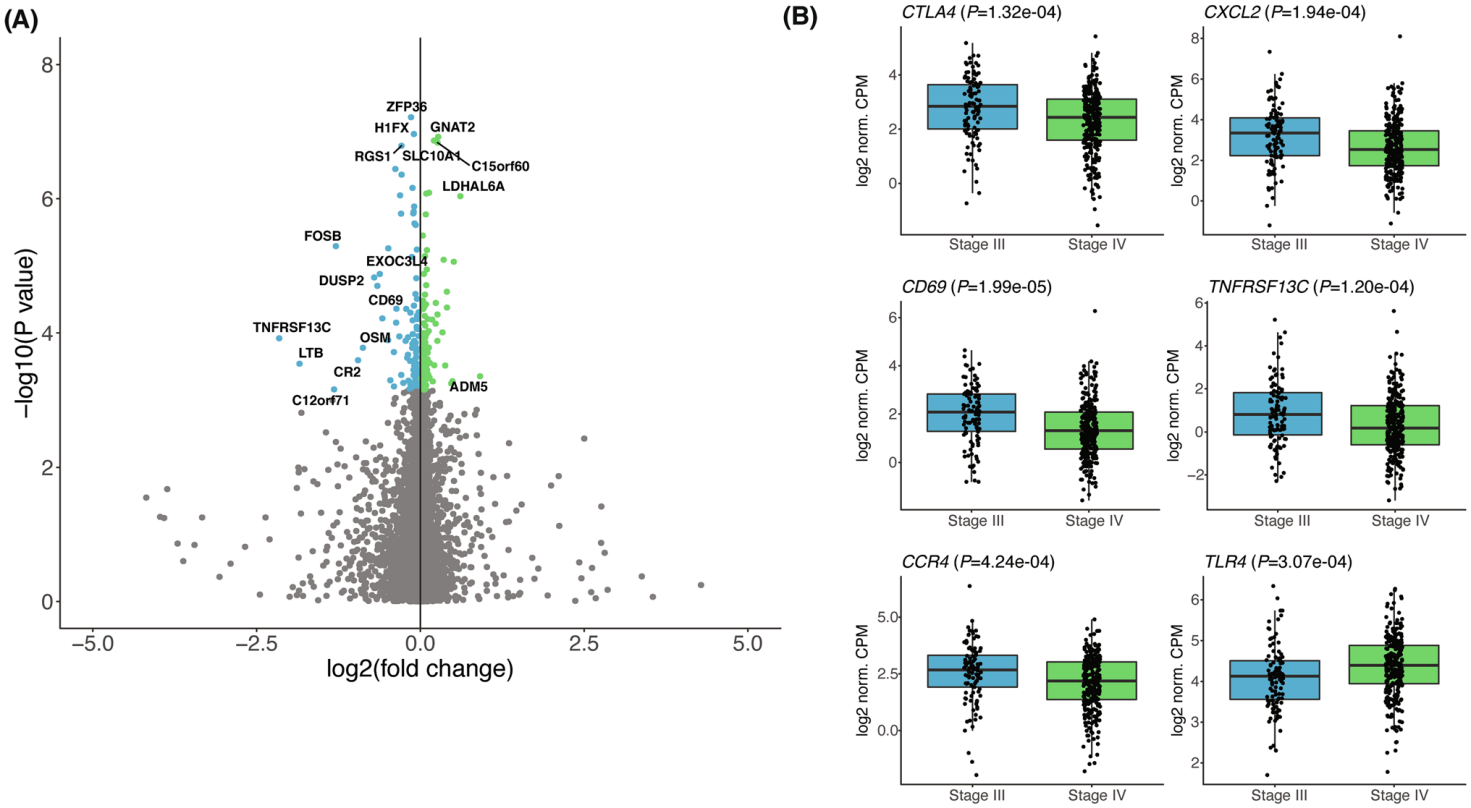
Transcriptome-wide differential expression analysis between stage III (*n* = 106) and stage IV (*n* = 285) tumors. **a** Volcano plot of gene expression differences between stage III and stage IV tumors. Each point represents a gene. Genes in blue had significantly increased expression (FDR 5%) in stage III tumors, while genes in green had significantly increased expression in stage IV tumors. Differentially expressed genes with an absolute fold change difference greater than > 1.5 are labeled. **b** Boxplots of selected significantly differentially expressed genes by tumor stage. Boxes represent the interquartile ranges, whiskers indicate the 95% confidence intervals. *CTLA4*, Cytotoxic T-Lymphocyte Associated Protein 4; *CXCL2*, C-X-C Motif Chemokine Ligand 2; *CD69*, Cluster of Differentiation 69; *TNFRSF13C*, Tumor Necrosis Factor Receptor Superfamily Member 13C; *CCR4*, C–C Motif Chemokine Receptor 4; *TLR4*, Toll-Like Receptor 4

We next characterized gene expression differences by stage across the transcriptome using a multivariable linear model. At a false discovery rate (FDR) of 5%, 205 genes were differentially expressed between stage III and IV tumors (Fig. 4a, Supplementary Table 1). Of those 205 genes, 91 (44%) had significantly increased expression in stage III tumors, and the remaining 114 in stage IV tumors. Boxplots of a manually selected collection of immune-related genes that were differentially expressed between the stages are presented in Fig. 4b. Most notably, *CTLA4* expression was significantly increased in stage III tumors (*P* = 1.32e-04), while there was no difference in expression observed for *CD274*, *PDCD1*, *LAG3*, *TIGIT*, or other ICB-related genes (Fig. 4b, Supplementary Fig. 2, Supplementary Table 3).

When stratified by *EGFR* status, 1,526 genes were found to be differentially expressed between *EGFR-*mutant and WT tumors, including *CCR4* (Supplementary Fig. 3) and *CD276* (Supplementary Table 4). Among the 1,526 differentially expressed genes identified, only 12 also significantly differed by stage (*C1orf111, CCR4, POU5F1, CDCA7, CYP4A11, CD83, BTN2A2, PBXIP1, OSGEP, DYNLL1, CD1A,* and *PIM3*), suggesting the expression differences identified between stage III and stage IV NSCLC tumors were not driven by *EGFR*-mutated tumors.

### Immune checkpoint biomarker expression between stage III and IV NSCLC tumors

While *CTLA4* was significantly differentially expressed by stage, no other selected ICB genes were (Supplementary Table 3), nor was *CTLA4* differentially expressed by *EGFR* (*n* = 53), *KRAS* (*n* = 160), *STK11* (*n* = 59), or *KEAP1* (*n* = 39) mutation status (Supplementary Table 4). The analysis of ICB gene expression by driver mutation status revealed mostly driver-specific differences. While *CD276* was significantly downregulated in *EGFR*-mutant tumors and upregulated in *KRAS*-mutant tumors, and *HAVCR1* (TIM-1) was upregulated in *STK11*- and *KEAP1*-mutant tumors, all other differentially expressed ICB genes were specific to one driver gene. This pattern held for *CD274* (PD-L1), which was downregulated in *STK11*-mutant tumors, but did not significantly differ in expression by *EGFR*, *KRAS*, or *KEAP1* mutation status.

### Gene set enrichment analysis (GSEA) of differentially expressed genes in stage III and IV NSCLC

**Fig. 5:**
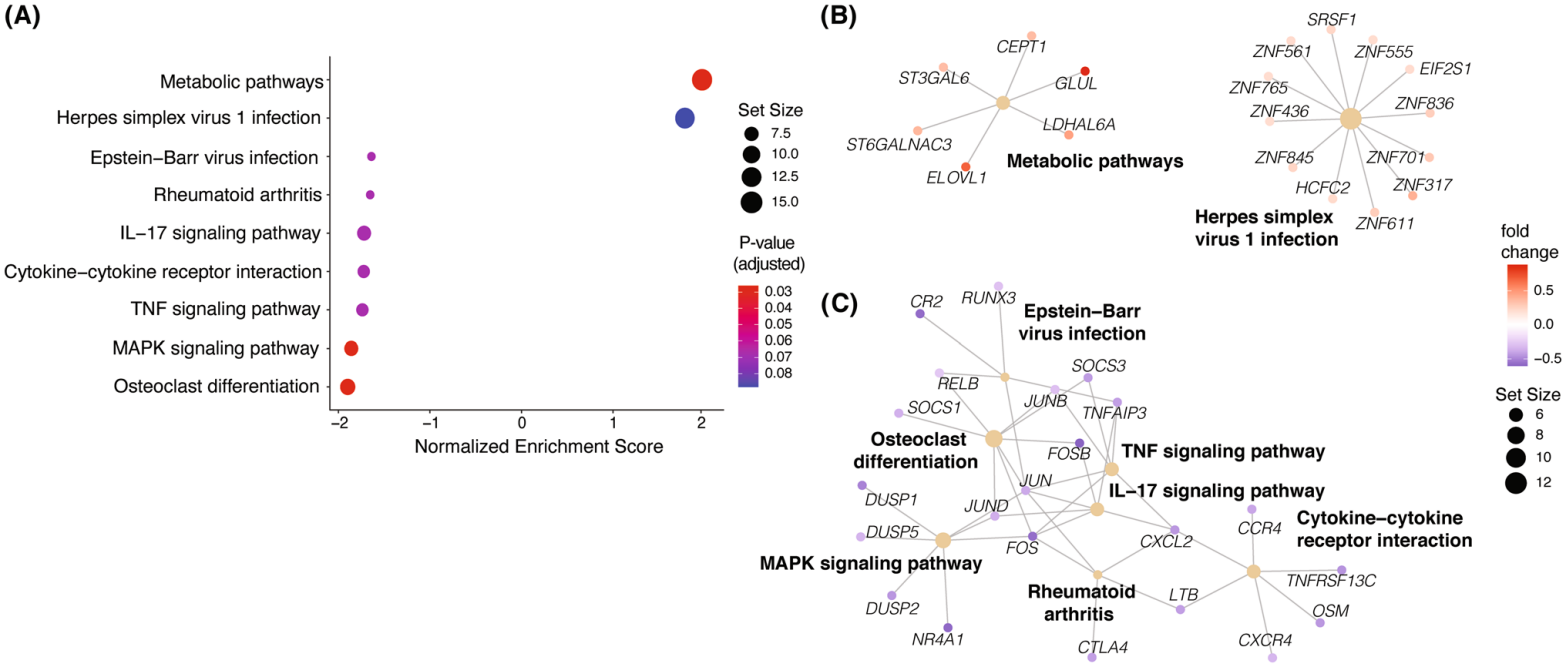
Gene set enrichment analysis (GSEA) of differentially expressed genes by tumor stage (stage III *n* = 106, stage IV *n* = 285). **a** Dot plot of KEGG pathway enrichment of pathways with adjusted *P* value < 0.1, *x*-axis indicates normalized enrichment score (NES). Dot color represents the adjusted *P* value of the gene set enrichment score, and dot size represents the number of genes included in each pathway. **b, c** Network visualization of genes in the listed pathways upregulated in stage IV tumors (**b**) or stage III tumors (**c**). Color of gene indicates the fold change between stage III and stage IV tumors, where a positive fold change (red) represents genes with increased expression in stage IV tumors, and negative fold change (blue) represents increased expression in stage III tumors. Dot size represents the number of genes evaluated in the pathway

To further characterize the gene expression differences between stage III and stage IV tumors, we performed gene set enrichment analysis (GSEA) on all genes with significant differential expression using the Kyoto Encyclopedia of Genes and Genomes (KEGG), Hallmark, and Gene Ontology (GO) gene sets. In stage IV tumors, the activity of metabolic pathways was significantly enriched (*P* = 0.004) (Fig. 5a-b). The herpes simplex virus 1 (HSV-1) pathway was also enriched in stage IV tumors (*P* = 0.014), which was mostly driven by expression of genes from the zinc finger protein (*ZNF*) family (Fig. 5c).

In contrast, several immune-related KEGG pathways were enriched in stage III tumors, including TNF signaling (*P* = 0.017), cytokine receptor interaction (*P* = 0.02), IL-17 signaling (*P* = 0.015), Epstein-Barr virus infection (*P* = 0.025), and rheumatoid arthritis (*P* = 0.024) (Fig. 5a). Underlying these pathways is a shared enrichment of several core pathway genes. Apart from the cytokine–cytokine receptor interaction pathway, all KEGG pathways enriched in stage III tumors shared relatively increased expression of genes from the *JUN* and *FOS* family. Meanwhile, IL-17 signaling, rheumatoid arthritis, and TNF signaling KEGG pathways were all linked to the cytokine–cytokine receptor interaction pathway through expression of *CXCL2* (Fig. 5c). A similar enrichment of immune regulatory pathways in stage III tumors was observed following Hallmark and GO GSEA (Supplementary Fig. 5 and Supplementary Fig. 6, respectively).

### Differences in immune cell proportions estimated from RNA-seq data

**Fig. 6:**
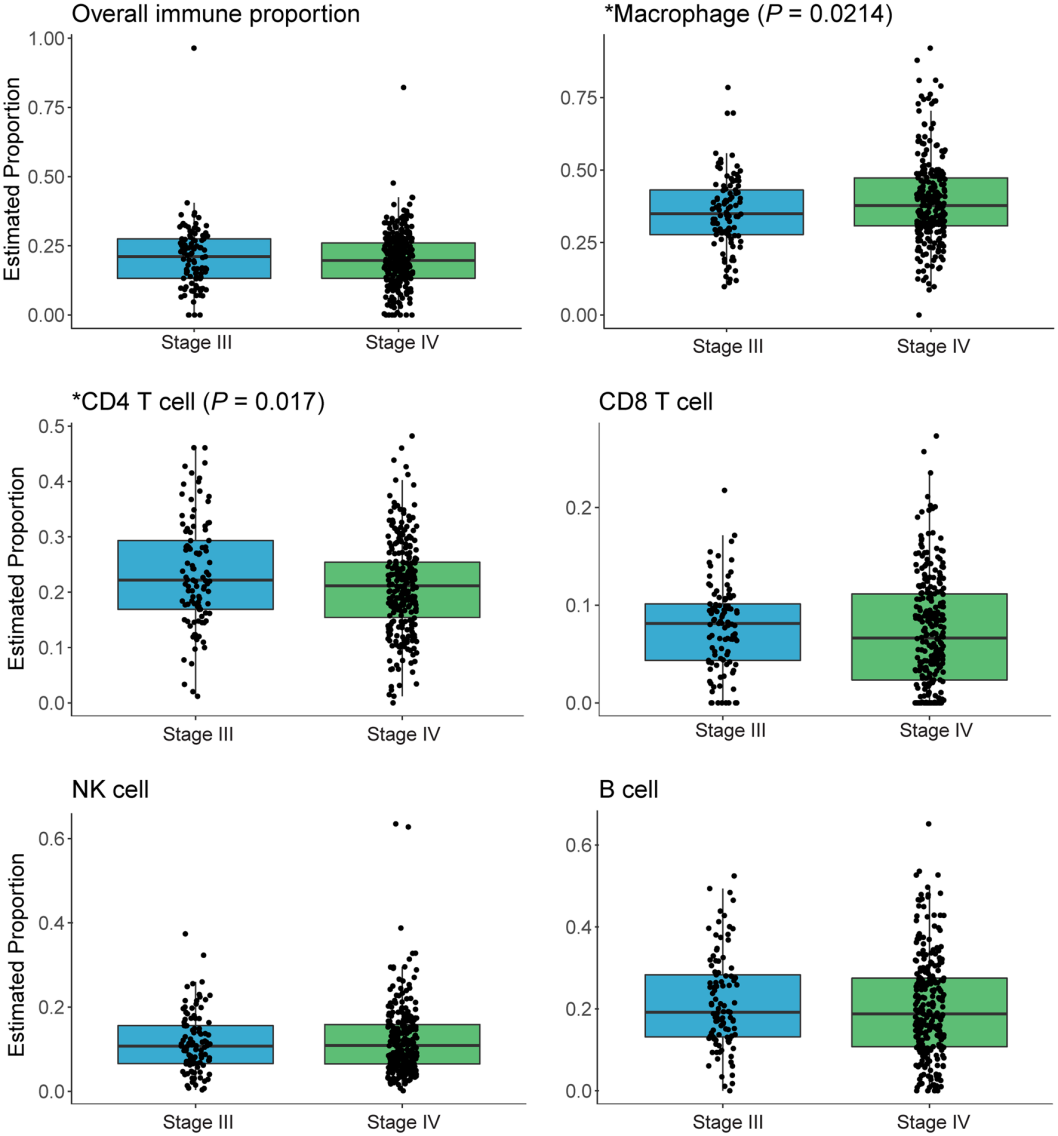
RNA-estimated immune compartment boxplots by stage (stage III *n* = 106, stage IV *n* = 285). Boxes represent the interquartile ranges, whiskers indicate the 95% confidence intervals. Macrophage and CD4 + T cell proportion significantly differ by stage, indicated by an asterisk

As differences in the immune cell composition of the TIME have been previously shown to correlate with survival and ICB treatment efficacy in patients with NSCLC, we next estimated the relative immune cell proportions from RNA-seq data in each tumor and compared between subsets of NSCLC tumors in the cohort [10, 17–19]. While overall estimated immune proportion did not differ by stage (Fig. 6) or *EGFR* status (Supplementary Fig. 7), there were differences observed in the proportions of individual cell types. Stage IV tumors had a significantly increased proportion of macrophages relative to stage III (*P* = 0.0214). Besides macrophage infiltration, differences were measured in CD4 + T cell quantities present in the TIME of each clinical subset. The proportion of CD4 + T cells was significantly higher in stage III tumors compared with stage IV (*P* = 0.017) (Fig. 6), and CD4 + T cells were relatively more abundant in *EGFR*-mutant tumors compared with WT (*P* = 0.0081) (Supplementary Fig. 7).

## Discussion

As concurrent treatment with ICBs in the first line has become standard care for stage III and IV NSCLC, clarifying distinctions between the TIMEs of these two groups may provide a new perspective on earlier-stage disease characteristics and spur opportunities to improve therapeutic strategies for both stages. Here, we assessed the transcriptomic landscape of stage III and IV NSCLC to establish a basic understanding of differences between these two populations and observed a variety of distinctions in gene expression, pathway activation, and estimated immune infiltration. To our knowledge, this direct comparison of RNA data between stage III and IV NSCLC has yet to be described in the literature.

While transcriptomics is of interest for future clinical applications, the current standard ICB biomarker is PD-L1 IHC. In this dataset, *CD274* gene expression from RNA-seq was highly correlated with PD-L1 TPS. Along with previously reported concordance between PD-L1 IHC and RNA-seq [20], these results suggest the viability of *CD274* RNA assessment as a proxy biomarker for PD-L1 IHC. Gauging PD-L1 levels initially from RNA-seq may be beneficial for patients as sequencing can simultaneously measure immunological signals and molecular characteristics often obtained through multiple approaches [21], as illustrated in our subsequent analyses of gene expression, pathway signaling, and estimated immune cell proportions. Although companion diagnostic IHC tests currently do not incorporate PD-L1 expression on TILs, it should be noted that TIL PD-L1 IHC was not correlated with overall *CD274* expression in this cohort. This is likely because the bulk RNA-seq data generated here were from a mixture of cells that were mainly tumor in origin. Future explorations of PD-L1 concordance with *CD274* could incorporate spatial transcriptomics to ameliorate the issues with RNA-seq of mixed cell populations [22, 23]. While the broad scope of whole-transcriptome RNA-seq is somewhat limiting in that sense [24], the same attribute is beneficial for unbiased large-scale assessments, which was highlighted in our differential expression analyses.

Interestingly, *CD274* (and, accordingly, PD-L1 IHC) expression was not significantly different between stage III and IV cancers, nor was the expression of *PDCD1* or *TIGIT*. Despite these findings and the substantial risk associated with inoperable stage III NSCLC, the recent breakthrough designation for dual blockade of TIGIT/PD-L1 was restricted to stage IV patients [25]. Along the same lines, combination of PD-L1 blockade with the CTLA4 inhibitor ipilimumab is currently not approved for stage III NSCLC patients, but *CTLA4* expression in this cohort was significantly higher in stage III tumors, although we acknowledge that expression of targets does not always correlate with more favorable responses and the potential for increased toxicity with dual CTLA4 blockade is a consideration. In addition to the differential expression observed in the RNA-based estimates of those clinically relevant biomarkers, we also identified differences in less substantiated therapeutic targets and prognostic markers. Stage III cancers had significantly higher expression of *CXCL2*, *CD69, CCR4*, and *TNFRsf13c*, all of which have been implicated as potential therapeutic targets in other malignancies [26–30], or in the case of *CCR4*, poor prognosis in lung cancer [31]. Conversely, *TLR4* expression was significantly higher in stage IV samples, consistent with a previous finding that *TLR4* overexpression was independently prognostic of poor overall and disease-free survival in NSCLC [32]. Considering that the differential expression patterns by stage described above were largely not observed in *EGFR* mutation status comparisons, these effects are likely independent from one another.

Along with differentially expressed genes identified, our evaluation of pathway activities also revealed distinctions between stage III and IV TIMEs. First, we observed significant enrichment of metabolic pathways in stage IV compared to stage III tumors. Increases in GLUL and ELOVL signaling were the most pronounced, which reflect increased metabolism of glutamine and lipids, respectively. These metabolic changes have been associated with cancer progression and suggest NSCLC metastasis is accompanied by an increased uptake in nutrients [33]. Stage III tumors had relatively increased activity in immune response pathways, on the other hand, especially those involved in T cell response. Again, many of the pathways enriched in stage III samples from this cohort have been implicated as potential therapeutic targets or prognostic indicators. IL-17, for example, is linked to NSCLC tumor progression via STAT3/NF-κB/Notch1 signaling in Th17 cells, and inhibition of the pathway was found to slow metastases [34, 35]. MAPK activity, which was also enriched in stage III tumors here, has been implicated as a therapeutic target and linked to the promotion of metastasis by IL-17 [36–38]. Accordingly, we found that the MAPK and IL-17 signaling pathways were connected through interaction with *Jun* and *Fos* genes in stage III tumors, which were also significantly upregulated. Moreover, previous studies have observed that exhausted T cells have lower expression of *Fos*, *Fosb*, and *Junb* [39, 40], supporting the conclusion that stage III tumors may have a more inflammatory (or at least less exhausted) TIME.

Lastly, estimates of immune cell proportions from RNA-seq data in the two stages showed a similar level of immune infiltration overall but a few key differences between individual cell type quantities. Most notably, CD4 + T cell quantities, which are crucial for anti-tumor immunity, were estimated as significantly higher in stage III tumors [41]. In fact, a recent study found that CD4 + T cells can enhance the activity of CD8 + cells and are correlated with response to ICB treatment in NSCLC [42].

Due to the broad, retrospective nature of this study, there are limitations in the findings. While age and sex were included as covariates in the differential expression analyses, we could not account for similar features that may have affected PD-L1 levels in the PD-L1 IHC correlation analyses. Race and ethnicity, for example, were not included due to sparsity of metadata from patient records. Germline and somatic variation, along with epigenetic effects, may have also influenced correlation between *CD274* expression and PD-L1 protein levels but were not considered here.

There are also several ICB-related factors to explore with next-generation sequencing that were not broached in these analyses. Relationships between circulating tumor cells [43], T cell receptor repertoires [44], and/or homologous recombination deficiency with ICB treatment have all been described [45], and further comparisons between stage III and IV TIMEs should consider these molecular characteristics. Regarding clinical characteristics, the data presented here are from pretreatment tumors. This selection was intentionally designed to control for treatment effects; however, many stage III-IV patients who receive ICB in real clinical scenarios have already undergone chemoradiotherapy. Given that neoadjuvant chemotherapy has been shown to activate immune response mechanisms in NSCLC [46], the same analysis in post-treatment tumors would be of benefit to the field. We also plan to extend the analyses conducted here to earlier stages, considering the recently published results in stage IA-IIIB patients [2, 3]. Specifically, we intend to integrate our findings with outcomes and treatment data to develop predictive transcriptomic signatures of response and/or resistance to ICBs in earlier-stage NSCLC.

Although the ultimate goal of future analyses is to inform clinical practice, the findings here are intended to serve as a base for ICB biomarker investigations and demonstrate the scope of possible TIME assessments through RNA-seq. These results add to the literature by demonstrating a direct comparison between the TIME in stage III and IV NSCLC, carry implications for further studies of ICB response in non-resectable stage III NSCLC, and provide data to guide further research of prognostic biomarkers and potential therapeutic targets in both stages.

## Methods

### Cohort selection

From the Tempus Database, 400 de-identified stage III or stage IV NSCLC patient health records and tumor samples were selected. Patients with neuroendocrine or pseudosarcomatous carcinoma histologies were excluded. Biopsies originating from the lung and/or airway were considered primary and included in the cohort. To reduce transcriptional variation due to medication and/or inclusion of unwanted tissue, samples were excluded if patients received prior therapy or if the biopsy was from a fine-needle aspirate procedure. Tumor stage was determined using information recorded within 30 days of the biopsy collection date, leaving a total of 391 samples for further analysis. Patient demographics and characteristics are presented in Table 1.

### Imputed smoking history

Recorded smoking status from clinical notes was missing in approximately 10% of patient records. To impute smoking status in these records, a transcriptome-based support vector machine (SVM) model was applied. The model was trained using 320 NSCLC patients with known smoking history. Using normalized counts of 18,570 genes as features, we selected the 500 most differentially expressed genes between known smokers (current smokers or ex-smokers) and nonsmokers. ‘*SelectKBest*’ from the ‘*sklearn*’ package in python was applied, using an ANOVA F-value as the metric. Our model was trained on an SVM with a linear kernel, and recursive feature elimination was applied to simplify. Cross-validation during recursive feature elimination was applied using the ‘*RFECV*’ function to select the best number of features. The final model achieved a cross-validation area under the curve (AUC) of 0.881 and a final validation AUC of 0.85.

### PD-L1 IHC

PD-L1 percentages in tumor cells (tumor proportion scores) and TILs were assessed by the 22C3 IHC assay, either through internal Tempus testing or results abstracted from de-identified patient records.

Correlations between PD-L1 IHC and *CD274* gene expression from RNA-seq were evaluated by the Spearman rank test. Stage-wise differences in mean values of PD-L1 IHC were assessed by the Mann–Whitney U test.

### Tempus xT panel sample processing and nucleic acid extraction

Expert pathologist assessment of overall tumor content and percent tumor cellularity as a ratio of tumor to normal nuclei verified specimens met a 20% threshold. Macrodissection was utilized as required to enrich specimens below the 20% threshold. Solid-tumor total nucleic acid was extracted from FFPE tissue sections using Chemagic 360 sample-specific extraction kits (PerkinElmer) and digested by proteinase K. RNA was purified from the total nucleic acid by DNase-I digestion.

### Tempus xT panel DNA and RNA library construction and sequencing

DNA sequencing of 648 genes and whole-transcriptome RNA sequencing were performed as previously described [47, 48]. Briefly, 50–300 nanograms (ng) of DNA for each tumor sample was mechanically sheared to an average size of 200 base pairs (bp) using a Covaris Ultrasonicator. DNA libraries were prepared using the KAPA Hyper Prep Kit, hybridized to the xT probe set, captured using Streptavidin-coated beads, and amplified with the KAPA HiFi Library Amplification Kit. For each tumor sample,100 ng of RNA was heat fragmented in the presence of magnesium to an average size of 200 bp. Library preps were hybridized to the xGEN Exome Research Panel v1.0 (Integrated DNA Technologies), and target recovery was performed using Streptavidin-coated beads, followed by amplification with the KAPA HiFi Library Amplification Kit. The amplified, target-captured DNA tumor library was sequenced using 2 × 126 bp paired-end reads to an average unique on-target depth of 500*x* (tumor) and 150*x* (normal) on an Illumina NovaSeq 6000. The amplified target-captured RNA tumor library was sequenced using 2 × 75 bp paired-end reads to an average of 50 million reads on an Illumina Novaseq 6000. Samples were further assessed for uniformity, with each sample required to have 95% of all targeted bp sequenced to a minimum depth of 300*x*.

Variant detection, visualization, and reporting were performed as previously described [47, 48].

### Tumor mutational burden

TMB was calculated by dividing the number of nonsynonymous mutations by the megabase size of the panel. All non-silent somatic coding mutations, including missense, indel and stop-loss variants with coverage greater than 100 × and an allelic fraction greater than 5%, were counted as non-synonymous mutations.

### RNA processing and analysis

Reads were pseudoaligned to hg19 using kallisto [49], and transcript counts were summed to genes for analysis. Genes located in mitochondrial DNA or the Y chromosome were removed, as were genes with < 1 counts per million in > 50% of patients, leaving 14,395 genes for downstream analysis. Data underwent trimmed means of *M*-value normalization (TMM) and voom transformation using the *R* packages *edgeR* and *limma* [50–52]. Principal components analysis (PCA) and Uniform Manifold Approximation and Projection (UMAP) using the R package *umap* were used on the normalized data to visualize the data and assess for potential confounders in the differential expression analysis.

Differentially expressed genes by stage were identified using *limma*, with age, sex, histology, tissue source, smoking history, and tumor purity included as covariates in a multivariable linear model. Significance was assessed using the methods of Benjamini and Hochberg after empirical Bayes moderation (function *eBayes()*) to better estimate gene-wise variability, and genes with a False Discovery Rate (FDR) < 5% were considered differentially expressed [53]. ICB gene list highlighted in supplementary tables is taken from supplementary table 1 from Auslander et al. [54].

We performed gene set enrichment analysis (GSEA) using the R package *ClusterProfiler* on differentially expressed genes (FDR < 0.05) [55]. Genes were ranked in descending order by their beta coefficient from the differential expression analysis. We performed GSEA in KEGG, Gene Ontology (GO), and Hallmark gene sets on the ranked genes to identify significantly enriched pathways using the respective functions and parameters: *gseKEGG*(parameters: minGSSize = 5, pvalueCutoff = 0.1); *GSEA*(parameters: TERM2GENE = c5, minGSSize = 5, pvalueCutoff = 0.1); *GSEA*(TERM2GENE = h, minGSSize = 5, pvalueCutoff = 0.1). To visualize the connectivity between enriched pathways, we used the *cnetplot()* function in ClusterProfiler to plot the pathway networks. All other figures were generated using *ggplot2* [56].

The relative proportions of immune cell subtypes were estimated using a support vector regression (SVR) model, as previously described [48].

### Statistical analysis

Differences in means were assessed using Student’s *t* test, unless noted. Correlations between PD-L1 IHC and other features were calculated using Pearson’s correlation. All differential expression results were determined using the *R* package *limm*a in a linear regression approach (see Methods above). Figure 1 is generated using CoMut [57], while all other figures were generated with *R* package *ggplot2* [56].

### Supplementary Information

Below is the link to the electronic supplementary material.

Supplementary file1 (PDF 3121 KB)

## Data Availability

All data relevant to the study are included in the article or uploaded as supplementary information. Further data are not available due to proprietary restrictions.
