# Supplementary material for: Comparison of the tumor immune microenvironment and checkpoint blockade biomarkers between stage III and IV non-small cell lung cancer
Source: Cancer Immunol Immunother. 2022 Jul 26;72(2):339–50. doi: 10.1007/s00262-022-03252-y (PMC9870967; doi:10.1007/s00262-022-03252-y)
Supplement: Supplementary file 1 — Supplementary file1 (PDF 3121 KB) [file 262_2022_3252_MOESM1_ESM.pdf]

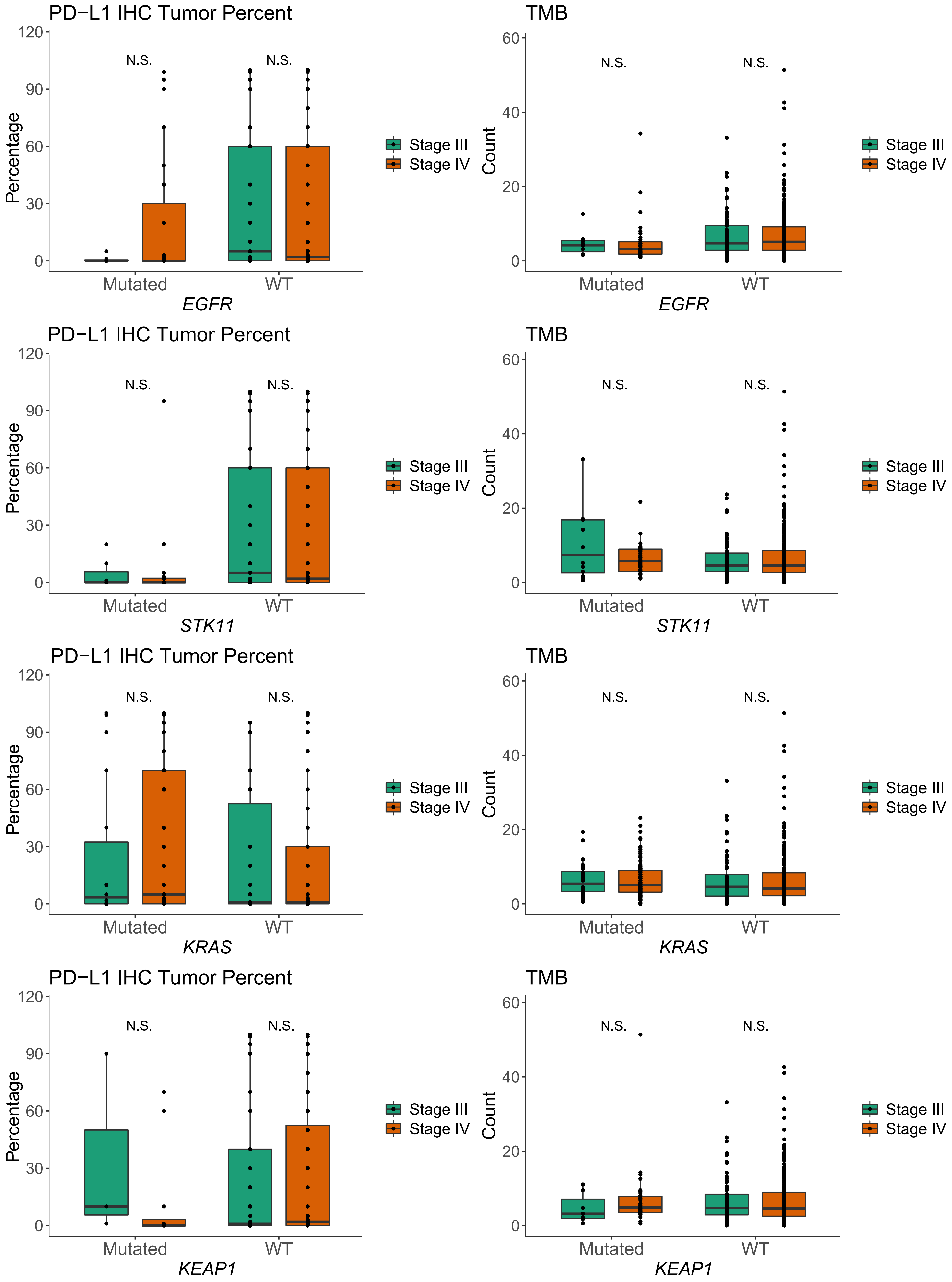

Supplementary Figure 1. Differences in oncogenic driver-mutant patients. Boxplots on the left show differences in PD-L1 tumor percent between EGFR-mutated (stage III n=7, stage IV n=27) vs. EGFR-wild-type (WT; stage III n=56, stage IV n=128), STK11-mutated (stage III n=7, stage IV n=19) vs. STK11-WT (stage III n=54, stage n=133), KRAS-mutated (stage III n=17, stage IV n=61) vs. KRAS-WT (stage III n=44, stage IV n=91), and KEAP1-mutated (stage III n=3, stage IV n=10) vs. KEAP1-WT (stage III n=58, stage IV n=142) tumors, colored by stage (stage III: green, stage IV: orange). Boxplots on the right show differences in TMB between EGFR-mutated (stage III n=11, stage IV n=42) vs. EGFR-wild-type (WT; stage III n=93, stage IV n=232), STK11-mutated (stage III n=13, stage IV n=44) vs. STK11-WT (stage III n=88, stage n=228), KRAS-mutated (stage III n=36, stage IV n=118) vs. KRAS-WT (stage III n=65, stage IV n=154), and KEAP1-mutated (stage III n=8, stage IV n=24) vs. KEAP1-WT (stage III n=93, stage IV n=248) tumors. No significant difference in either PD-L1 or TMB were identified between stage III and stage IV tumors after stratifying by the mutation status of those oncogenic drivers.

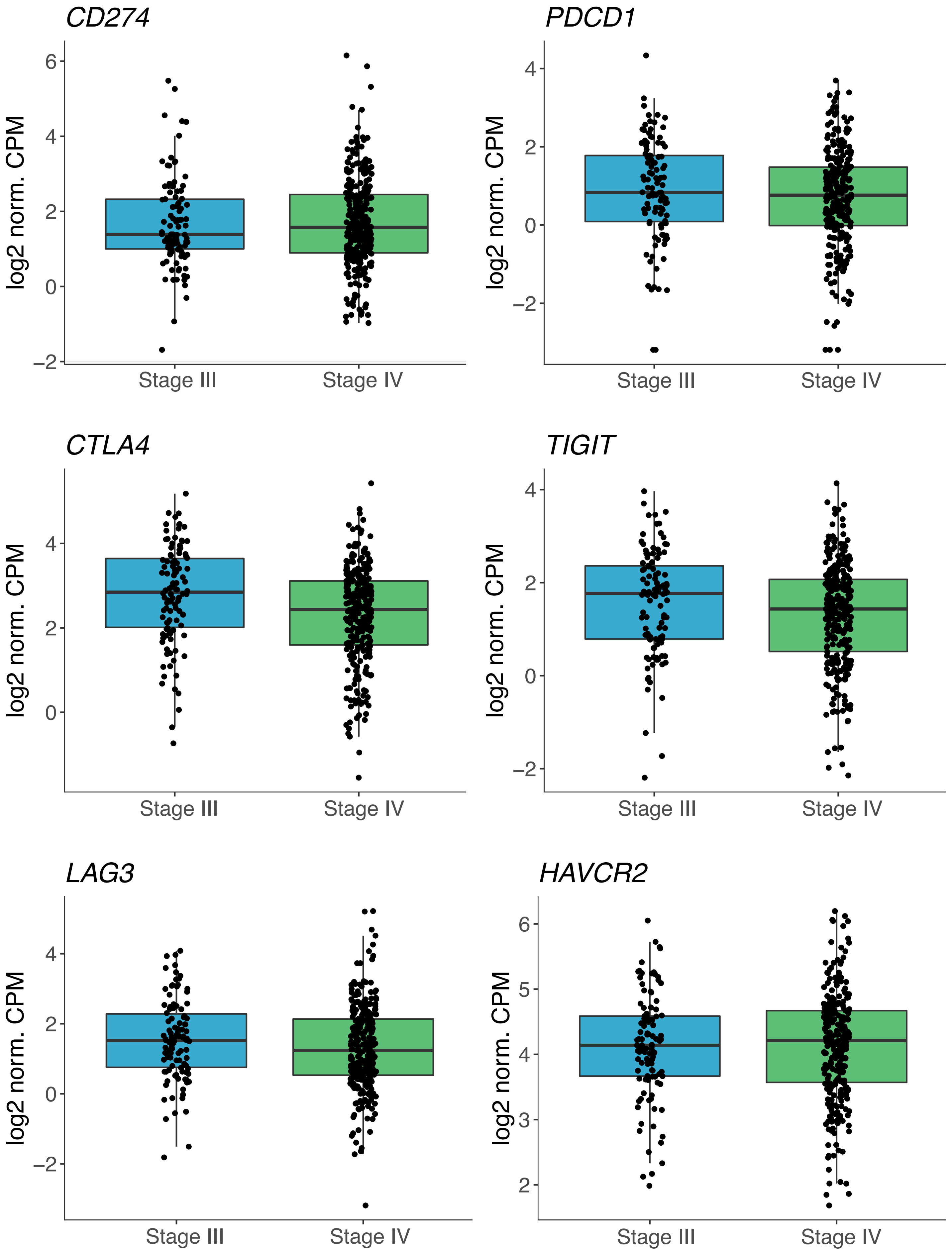

Supplementary Figure 2. Immune checkpoint gene expression boxplots by tumor stage (stage III n=106, stage IV n=285). Boxes represent the interquartile ranges, whiskers indicate the 95% confidence intervals. CD274, Programmed Cell Death 1 Ligand; PDCD1, Programmed Cell Death 1; CTLA4, Cytotoxic T-Lymphocyte Associated Protein 4; TIGIT, T Cell Immunoreceptor with Ig and ITIM Domain; LAG3, Lymphocyte Activating 3; HAVCR2, T-Cell Immunoglobulin And Mucin Domain-Containing Protein 3. Of the genes above, only the expression of CTLA4 significantly differed by stage (P=1.32e-04).

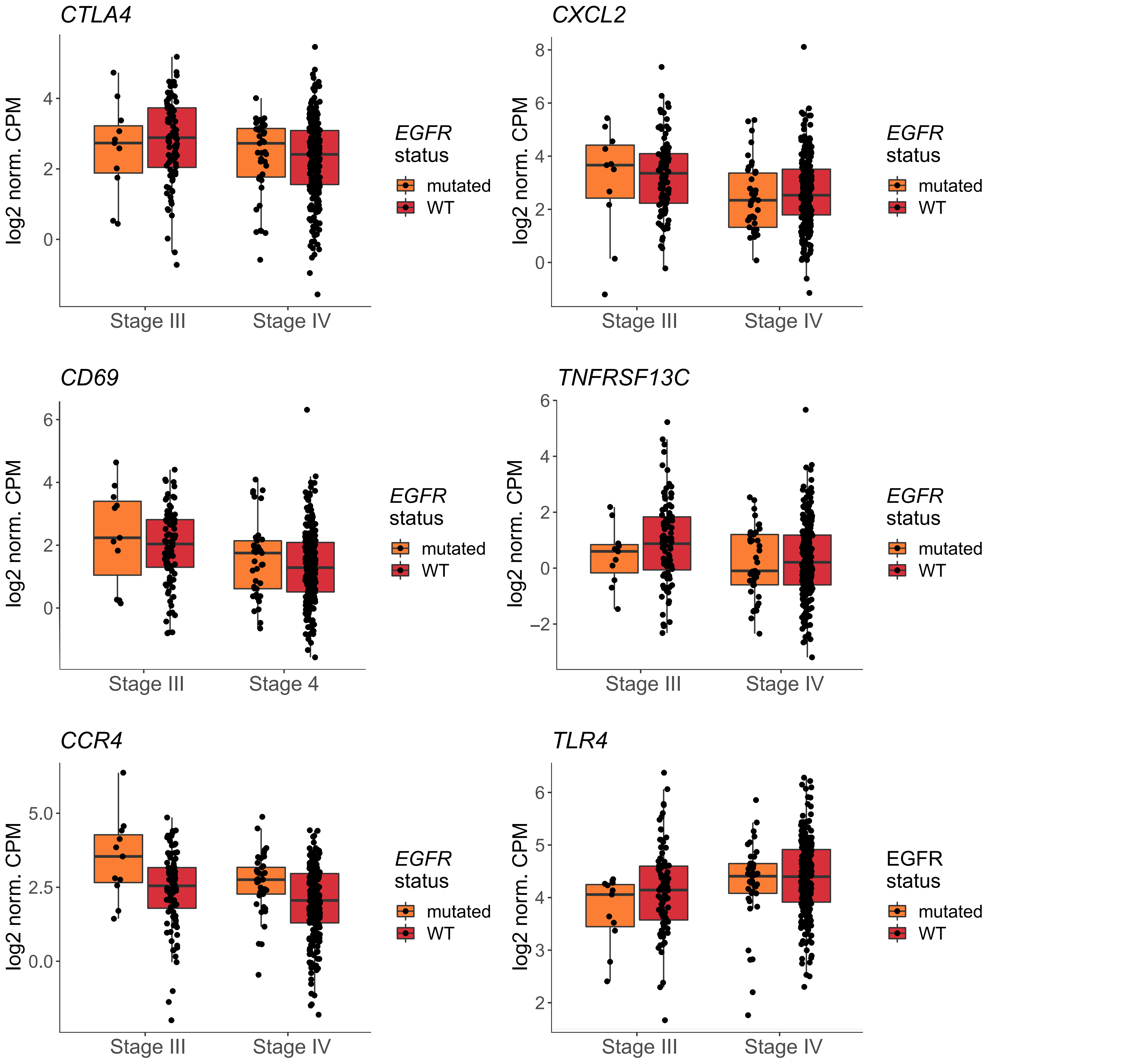

Supplementary Figure 3. Expression of genes differentially expressed by tumor stage by EGFR mutation status (mutated=pathogenic mutation in EGFR, stage III n=11 and stage IV n=42; WT= wild-type EGFR, n=338). Boxes represent the interquartile ranges, whiskers indicate the 95% confidence intervals. CTLA4, Cytotoxic T-Lymphocyte Associated Protein 4; CXCL2, C-X-C Motif Chemokine Ligand 2; CD69, Cluster of Differentiation 69; TNFRSF13C, Tumor Necrosis Factor Receptor Superfamily Member 13C; CCR4, C-C Motif Chemokine Receptor 4; TLR4, Toll Like Receptor 4. CCR4 was differentially expressed (FDR 5% significance threshold) by stage (*P*=6.50e-05) and EGFR status (*P*=2.11e-05). For all other genes, expression difference by EGFR status was not significant.

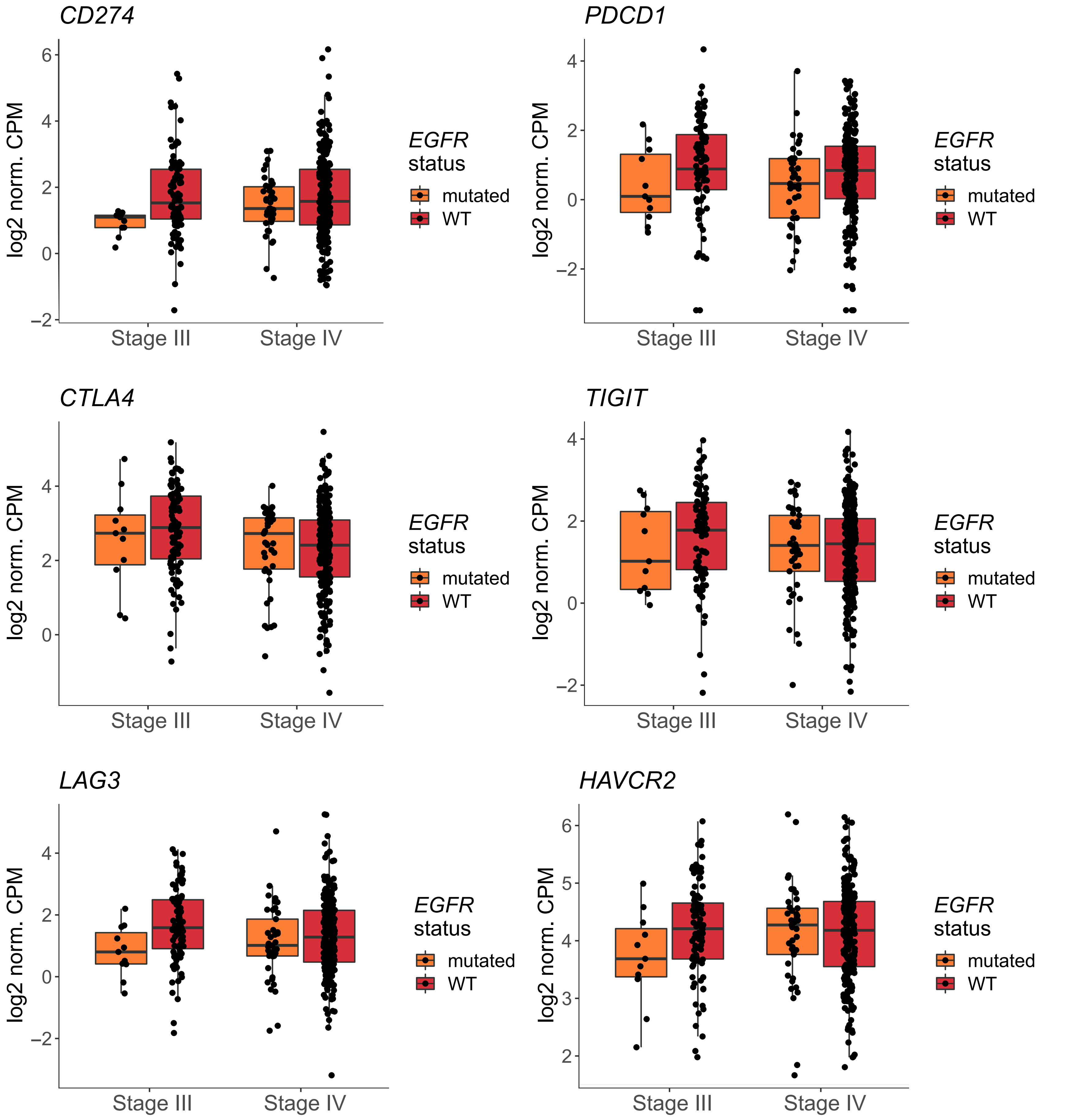

Supplementary Figure 4. Immune checkpoint gene expression boxplots by stage and EGFR mutation status (mutated=pathogenic mutation in EGFR, stage III n=11 and stage IV n=42; WT= wild-type EGFR, n=338). Boxes represent the interquartile ranges, whiskers indicate the 95% confidence intervals. CD274, Programmed Cell Death 1 Ligand; PDCD1, Programmed Cell Death 1; CTLA4, Cytotoxic T-Lymphocyte Associated Protein 4; TIGIT, T Cell Immunoreceptor with Ig And ITIM Domain; LAG3, Lymphocyte Activating 3; HAVCR2, T-Cell Immunoglobulin And Mucin Domain-Containing Protein 3. Expression of CTLA4 significantly differed by stage (*P*=1.32e-04) but none of the genes shown had significantly different expression levels by EGFR mutation status.

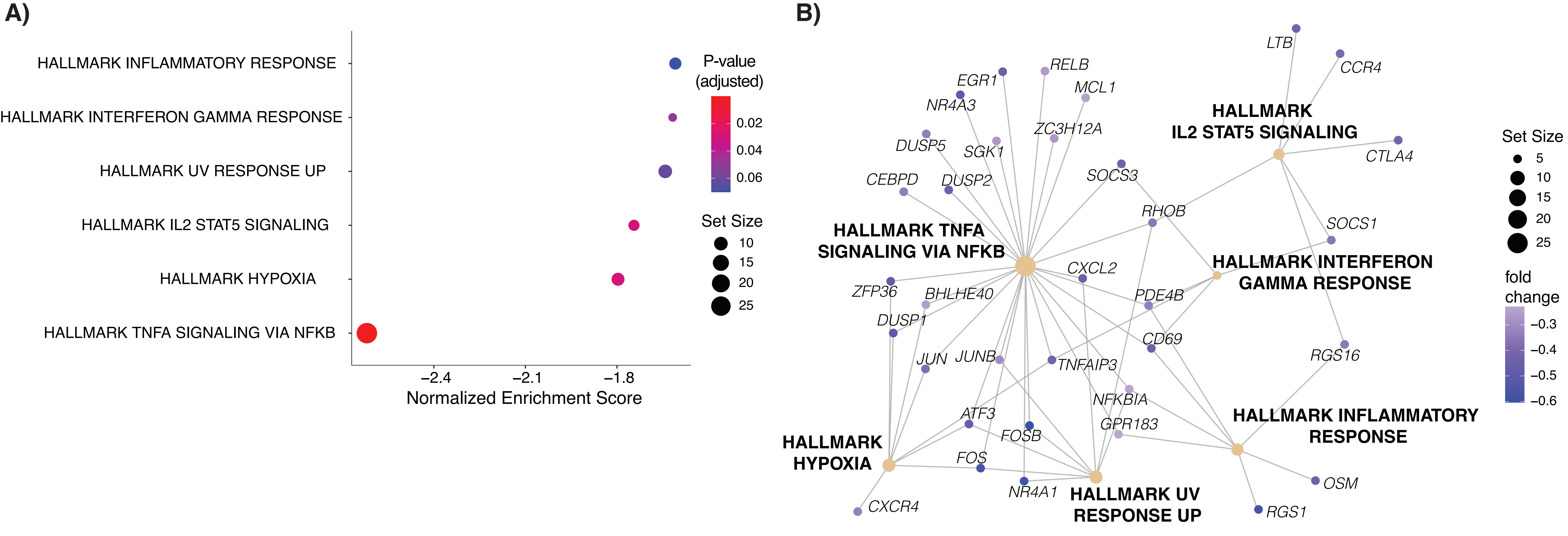
A)
HALLMARK INFLAMMATORY RESPONSE
HALLMARK INTERFERON GAMMA RESPONSE
HALLMARK UV RESPONSE UP
HALLMARK IL2 STAT5 SIGNALING
HALLMARK HYPOXIA
HALLMARK TNFA SIGNALING VIA NFKB
−2.4
−2.1
−1.8
Normalized Enrichment Score
P-value (adjusted)
0.02
0.04
0.06
Set Size
10
15
20
25
B)
HALLMARK IL2 STAT5 SIGNALING
HALLMARK TNFA SIGNALING VIA NFKB
HALLMARK INTERFERON GAMMA RESPONSE
HALLMARK INFLAMMATORY RESPONSE
HALLMARK HYPOXIA
HALLMARK UV RESPONSE UP
LTB
CCR4
RELB
EGR1
MCL1
NR4A3
ZC3H12A
DUSP5
SGK1
CTLA4
CEBPD
DUSP2
SOCS3
RHOB
SOCS1
CXCL2
ZFP36
BHLHE40
PDE4B
DUSP1
CD69
JUN
JUNB
TNFAIP3
RGS16
NFKBIA
ATF3
GPR183
FOSB
FOS
NR4A1
OSM
CXCR4
RGS1
Set Size
5
10
15
20
25
fold change
−0.3
−0.4
−0.5
−0.6

Supplementary Figure 5. Hallmark gene set enrichment analysis (GSEA) of differentially expressed genes by tumor stage (stage III n=106, stage IV n=285). (A) Dotplot of Hallmark pathway enrichment of pathways with adjusted P-value <0.1, x-axis indicates normalized enrichment score (NES). Color of dot represents the adjusted P-value of the GSEA score. (B) Network visualization of genes in the listed pathways upregulated in stage III tumors. Color of gene represents the fold change between stage III and stage IV tumors, where a positive fold change (red) indicates genes with increased expression in stage IV tumors, and negative fold change (blue) represents increased expression in stage III tumors.

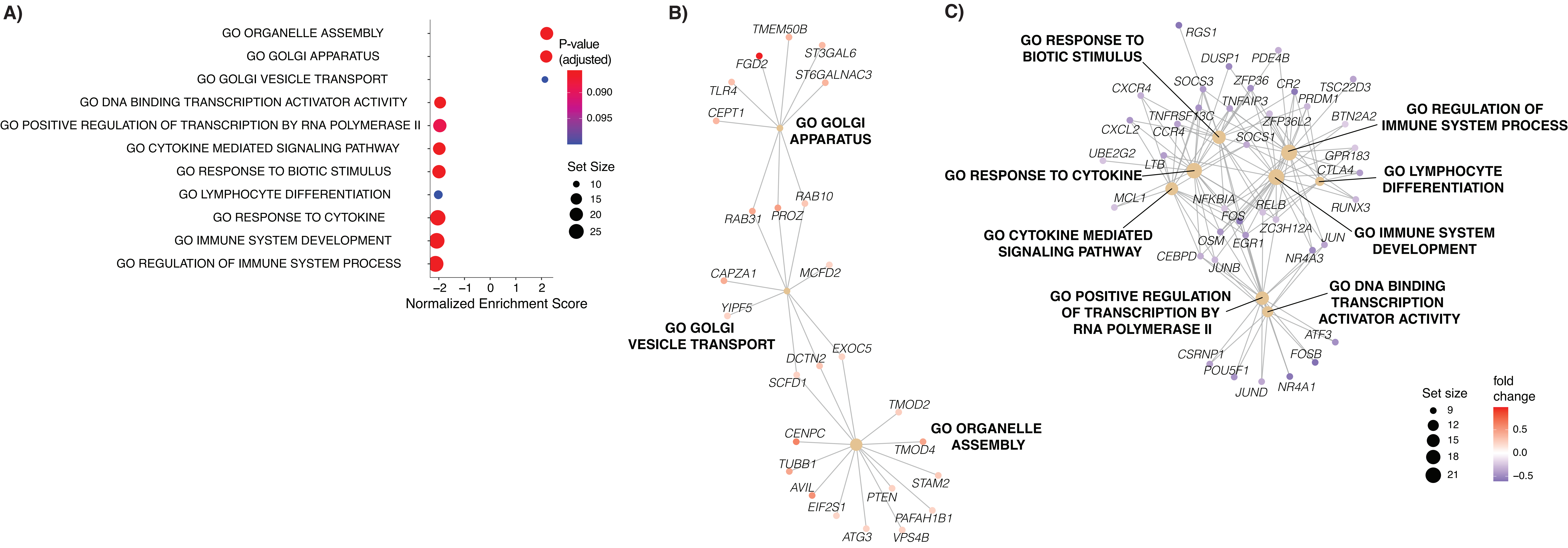
A)
GO ORGANELLE ASSEMBLY
GO GOLGI APPARATUS
GO GOLGI VESICLE TRANSPORT
GO DNA BINDING TRANSCRIPTION ACTIVATOR ACTIVITY
GO POSITIVE REGULATION OF TRANSCRIPTION BY RNA POLYMERASE II
GO CYTOKINE MEDIATED SIGNALING PATHWAY
GO RESPONSE TO BIOTIC STIMULUS
GO LYMPHOCYTE DIFFERENTIATION
GO RESPONSE TO CYTOKINE
GO IMMUNE SYSTEM DEVELOPMENT
GO REGULATION OF IMMUNE SYSTEM PROCESS
−2 −1 0 1 2
Normalized Enrichment Score
P-value (adjusted)
0.090
0.095
Set Size
10
15
20
25
B)
TMEM50B
ST3GAL6
FGD2
ST6GALNAC3
TLR4
CEPT1
GO GOLGI APPARATUS
RAB10
RAB31
PROZ
CAPZA1
MCFD2
YIPF5
GO GOLGI VESICLE TRANSPORT
EXOC5
DCTN2
SCFD1
TMOD2
GO ORGANELLE ASSEMBLY
CENPC
TMOD4
TUBB1
STAM2
AVIL
PTEN
EIF2S1
PAFAH1B1
ATG3
VPS4B
C)
RGS1
GO RESPONSE TO BIOTIC STIMULUS
DUSP1
PDE4B
SOCS3
ZFP36
CXCR4
CR2
TSC22D3
TNFAIP3
PRDM1
TNFRSF13C
GO REGULATION OF IMMUNE SYSTEM PROCESS
BTN2A2
ZFP36L2
CXCL2
CCR4
SOCS1
UBE2G2
GPR183
LTB
CTLA4
GO RESPONSE TO CYTOKINE
GO LYMPHOCYTE DIFFERENTIATION
MCL1
NFKBIA
RELB
RUNX3
FOS
ZC3H12A
GO CYTOKINE MEDIATED SIGNALING PATHWAY
OSM
EGR1
JUN
GO IMMUNE SYSTEM DEVELOPMENT
CEBPD
NR4A3
JUNB
GO DNA BINDING TRANSCRIPTION ACTIVATOR ACTIVITY
GO POSITIVE REGULATION OF TRANSCRIPTION BY RNA POLYMERASE II
ATF3
CSRNP1
FOSB
POU5F1
JUND
NR4A1
Set size
9
12
15
18
21
fold change
0.5
0.0
−0.5

Supplementary Figure 6. Gene ontology (GO) Gene set enrichment analysis (GSEA) of differentially expressed genes by tumor stage (stage III n=106, stage IV n=285). (A) Dotplot of GO pathway enrichment of pathways with adjusted P-value <0.1, x-axis indicates normalized enrichment score (NES). Dot color represents the adjusted P-value of the gene set enrichment score. Network visualization of genes in the listed pathways upregulated in stage IV tumors (B) or stage III tumors (C). Color of gene represents the fold change between stage III and stage IV tumors, where a positive fold change (red) indicates genes with increased expression in stage IV tumors, and negative fold change (blue) represents increased expression in stage III tumors.

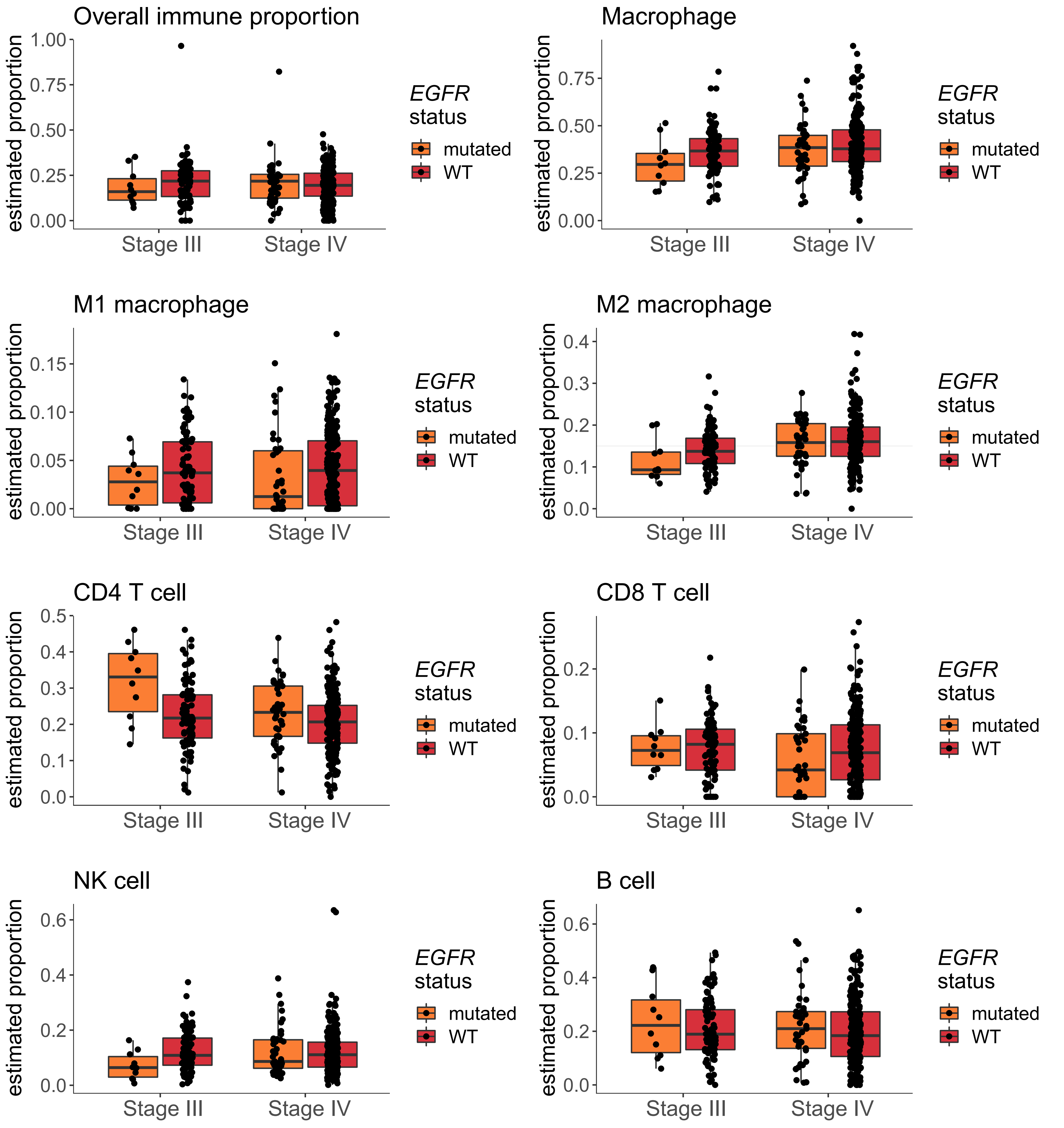

Supplementary Figure 7. RNA-estimated immune compartment boxplots by stage and EGFR mutation status (mutated=pathogenic mutation in EGFR, stage III n=11 and stage IV n=42; WT= wild-type EGFR, n=338). Boxes represent the interquartile ranges, whiskers indicate the 95% confidence intervals. Macrophage and CD4+ T cell proportions significantly differed by stage. CD4+ T cell proportions also significantly differed by EGFR status.

Supplementary Table 1.
List of differentially expressed genes by tumor stage (FDR 5%) with regression P-value and direction of effect (stage III n=106, stage IV n=285).

Gene Symbol	P-value	Direction of Effect (increased expression)
ZFP36	6.09E-08	stage III
H1FX	1.08E-07	stage III
RGS1	1.62E-07	stage III
NR4A3	3.60E-07	stage III
NR4A1	4.38E-07	stage III
SMIM7	6.89E-07	stage III
ATF3	8.91E-07	stage III
JUND	1.31E-06	stage III
CSRNP1	1.56E-06	stage III
TNFAIP3	1.65E-06	stage III
DUSP1	1.67E-06	stage III
ITPKC	2.33E-06	stage III
DCUN1D3	2.46E-06	stage III
FOSB	5.09E-06	stage III
C11orf96	5.49E-06	stage III
MCL1	5.73E-06	stage III
JUN	7.40E-06	stage III
EXOC3L4	1.32E-05	stage III
DUSP2	1.49E-05	stage III
FAM120AOS	1.53E-05	stage III
CD69	1.99E-05	stage III
RELB	2.63E-05	stage III
ITPRIP	3.12E-05	stage III
PDE4B	3.93E-05	stage III
FOS	4.37E-05	stage III
GRASP	4.40E-05	stage III

Gene Symbol	P-value	Direction of Effect (increased expression)
GNAT2	1.19E-07	stage IV
SLC10A1	1.36E-07	stage IV
C15orf60	1.46E-07	stage IV
ZNF317	8.14E-07	stage IV
UBE2E1	8.38E-07	stage IV
LDHAL6A	9.17E-07	stage IV
NFYB	1.71E-06	stage IV
DDX19A	3.54E-06	stage IV
CAPZA1	5.83E-06	stage IV
CSNK1G1	7.17E-06	stage IV
LIN37	8.13E-06	stage IV
PROZ	8.71E-06	stage IV
TPRKB	1.14E-05	stage IV
EIF5	1.32E-05	stage IV
DIS3L	1.95E-05	stage IV
CRYBA1	2.43E-05	stage IV
STAM2	2.69E-05	stage IV
UFSP2	3.31E-05	stage IV
ELOVL1	3.58E-05	stage IV
EXOC5	3.75E-05	stage IV
ZNF561	3.89E-05	stage IV
C18orf21	3.90E-05	stage IV
CRYBG3	4.11E-05	stage IV
TMOD4	4.19E-05	stage IV
ZNF701	4.40E-05	stage IV
AVIL	5.33E-05	stage IV

PSPC1	4.95E-05	stage III
SOCS3	5.00E-05	stage III
HIST1H1E	5.56E-05	stage III
XIRP1	6.09E-05	stage III
STRADA	6.24E-05	stage III
RGPD5	6.50E-05	stage III
C1orf111	7.05E-05	stage III
MED26	8.13E-05	stage III
HIST1H2BD	8.68E-05	stage III
DHDDS	8.81E-05	stage III
CYR61	1.04E-04	stage III
MRPL20	1.11E-04	stage III
SOCS1	1.13E-04	stage III
APOLD1	1.16E-04	stage III
TNFRSF13C	1.20E-04	stage III
TMEM160	1.28E-04	stage III
CTLA4	1.32E-04	stage III
RHOB	1.32E-04	stage III
SGK1	1.40E-04	stage III
GNB2L1	1.46E-04	stage III
ZC3H12A	1.53E-04	stage III
OSM	1.67E-04	stage III
ADAMTS1	1.67E-04	stage III
DDX5	1.72E-04	stage III
CDK11B	1.80E-04	stage III
CXCL2	1.94E-04	stage III
ZFP36L2	1.97E-04	stage III
RGS16	2.12E-04	stage III
CNNM3	2.29E-04	stage III
POU5F1	2.36E-04	stage III
PIM3	2.49E-04	stage III

ZNF555	5.60E-05	stage IV
TRIQK	6.23E-05	stage IV
RAB31	6.35E-05	stage IV
MCFD2	7.19E-05	stage IV
ADORA3	7.30E-05	stage IV
RAB10	7.82E-05	stage IV
CEPT1	9.39E-05	stage IV
C14orf183	9.83E-05	stage IV
ZNF845	1.00E-04	stage IV
TXNL4B	1.03E-04	stage IV
AGPAT6	1.05E-04	stage IV
LMBRD1	1.12E-04	stage IV
ZNF836	1.15E-04	stage IV
ZHX1	1.27E-04	stage IV
UBE2B	1.27E-04	stage IV
THAP1	1.29E-04	stage IV
AIDA	1.29E-04	stage IV
ATG3	1.31E-04	stage IV
FSD2	1.32E-04	stage IV
FAM63B	1.39E-04	stage IV
CCNL1	1.45E-04	stage IV
ZNF611	1.54E-04	stage IV
C14orf28	1.66E-04	stage IV
MRPL13	1.67E-04	stage IV
C8orf59	1.69E-04	stage IV
GTPBP10	1.74E-04	stage IV
DENND4C	1.77E-04	stage IV
CLPX	1.86E-04	stage IV
PP2D1	1.98E-04	stage IV
PTEN	2.01E-04	stage IV
DCTN2	2.06E-04	stage IV

CR2	2.56E-04	stage III
EGR1	2.63E-04	stage III
PRDM1	2.85E-04	stage III
LTB	2.89E-04	stage III
PGS1	2.93E-04	stage III
GPR183	3.09E-04	stage III
RUNX3	3.16E-04	stage III
RNF122	3.18E-04	stage III
RNF19B	3.44E-04	stage III
HIST1H4K	3.56E-04	stage III
ZFAND5	3.63E-04	stage III
EXOSC7	3.77E-04	stage III
CEBPD	4.02E-04	stage III
SNRNP70	4.06E-04	stage III
CCR4	4.24E-04	stage III
HIST1H2AK	4.27E-04	stage III
SIRT3	4.75E-04	stage III
TUT1	4.99E-04	stage III
CDHR1	5.09E-04	stage III
UBE2G2	5.16E-04	stage III
NFKBIA	5.24E-04	stage III
BHLHE40	5.34E-04	stage III
JUNB	5.48E-04	stage III
HUS1B	5.57E-04	stage III
GPR89C	5.60E-04	stage III
CXCR4	5.80E-04	stage III
MAB21L2	5.84E-04	stage III
CATSPER2	6.30E-04	stage III
MOGS	6.34E-04	stage III
OSGEP	6.42E-04	stage III
TSC22D3	6.68E-04	stage III

RYK	2.27E-04	stage IV
GNPDA2	2.34E-04	stage IV
UBA3	2.41E-04	stage IV
WDR1	2.42E-04	stage IV
HCFC2	2.47E-04	stage IV
YIPF5	2.63E-04	stage IV
ITFG1	2.74E-04	stage IV
ST3GAL6	2.80E-04	stage IV
ZNF436	2.85E-04	stage IV
TMEM50B	2.91E-04	stage IV
VPS4B	2.94E-04	stage IV
DNAJA2	2.97E-04	stage IV
ST6GALNAC3	3.04E-04	stage IV
GLUL	3.07E-04	stage IV
TLR4	3.07E-04	stage IV
ZNF654	3.11E-04	stage IV
AP1G1	3.17E-04	stage IV
FEM1B	3.18E-04	stage IV
SRSF1	3.43E-04	stage IV
NCOA5	3.44E-04	stage IV
CD164	3.50E-04	stage IV
OPN1SW	3.58E-04	stage IV
CNOT1	3.60E-04	stage IV
EYS	3.65E-04	stage IV
CHURC1	3.88E-04	stage IV
RBMS2	3.98E-04	stage IV
TUBB1	4.19E-04	stage IV
EMC2	4.27E-04	stage IV
PSEN1	4.35E-04	stage IV
CNEP1R1	4.38E-04	stage IV
BMI1	4.38E-04	stage IV

DUSP5	6.83E-04	stage III
C12orf71	6.97E-04	stage III
BTN2A2	7.08E-04	stage III

EFR3A	4.39E-04	stage IV
ADM5	4.43E-04	stage IV
TMEM86A	4.65E-04	stage IV
RNF130	4.71E-04	stage IV
WWP1	4.82E-04	stage IV
SCFD1	4.85E-04	stage IV
TMOD2	4.87E-04	stage IV
MTRF1L	4.99E-04	stage IV
ZNF280D	5.07E-04	stage IV
STXBP5	5.20E-04	stage IV
C12orf4	5.23E-04	stage IV
FGD2	5.26E-04	stage IV
SLC30A9	5.29E-04	stage IV
CENPC	5.69E-04	stage IV
IL6ST	5.72E-04	stage IV
NCKAP1	5.80E-04	stage IV
PAFAH1B1	5.82E-04	stage IV
C5orf22	6.04E-04	stage IV
CMTR2	6.14E-04	stage IV
UGP2	6.18E-04	stage IV
KPNA4	6.55E-04	stage IV
USP53	6.78E-04	stage IV
CUL4B	6.80E-04	stage IV
EIF2S1	6.88E-04	stage IV
LATS1	6.93E-04	stage IV
ZNF765	7.06E-04	stage IV

Supplementary Table 2.

Genes with differences in mutation frequency (P<0.1) between stage III and stage IV patients (stage III n=106, stage IV n=285).

	**Stage III (n=106)**	**Stage IV (n=284)**	**P-Value**	**Adjusted P-Value (Bonferroni)**
RNF139	5 (4.7%)	0 (0%)	0.00	0.04
ERG	5 (4.7%)	1 (0.4%)	0.01	0.21
MUTYH	5 (4.7%)	1 (0.4%)	0.01	0.21
FOXO3	6 (5.7%)	3 (1.1%)	0.01	0.46
FANCD2	7 (6.6%)	5 (1.8%)	0.02	0.67
PHF6	4 (3.8%)	1 (0.4%)	0.02	0.66
RBM10	21 (19.8%)	30 (10.6%)	0.03	0.80
KDM6A	6 (5.7%)	4 (1.4%)	0.03	0.89
ERRFI1	4 (3.8%)	2 (0.7%)	0.05	1.00
FOXP1	4 (3.8%)	2 (0.7%)	0.05	1.00
PRCC	4 (3.8%)	2 (0.7%)	0.05	1.00
ABCB1	4 (3.8%)	30 (10.6%)	0.06	1.00
CTCF	3 (2.8%)	1 (0.4%)	0.06	1.00
HLA-F	3 (2.8%)	1 (0.4%)	0.06	1.00
TCL1A	3 (2.8%)	1 (0.4%)	0.06	1.00
SETD2	14 (13.2%)	19 (6.7%)	0.06	1.00
STAT5A	5 (4.7%)	4 (1.4%)	0.07	1.00
FBXW7	0 (0%)	10 (3.5%)	0.07	1.00
HLA-DRB1	0 (0%)	10 (3.5%)	0.07	1.00
TNFAIP3	0 (0%)	10 (3.5%)	0.07	1.00
MAFB	2 (1.9%)	0 (0%)	0.07	1.00
RIT1	2 (1.9%)	0 (0%)	0.07	1.00

ASXL1	9 (8.5%)	10 (3.5%)	0.08	1.00
CTNNB1	9 (8.5%)	10 (3.5%)	0.08	1.00
BCR	1 (0.9%)	14 (4.9%)	0.08	1.00
DICER1	1 (0.9%)	14 (4.9%)	0.08	1.00
FANCB	1 (0.9%)	14 (4.9%)	0.08	1.00
SMARCA1	1 (0.9%)	15 (5.3%)	0.08	1.00
RUNX1T1	11 (10.4%)	14 (4.9%)	0.09	1.00
MTOR	2 (1.9%)	20 (7.0%)	0.09	1.00
CYP1B1	4 (3.8%)	3 (1.1%)	0.09	1.00
SRSF2	4 (3.8%)	3 (1.1%)	0.09	1.00

Supplementary Table 3. Differential expression results of immune checkpoint genes by tumor stage, unadjusted P-value shown (stage III n=106, stage IV n=285). Asterisk (*) indicates the gene was significantly differentially expressed (FDR 5%) in a transcriptome-wide analysis, with direction of effect noted in "If DE".

**ICB Gene**	**P-value**	**If DE, increased in:**
BTLA	2.85E-01	
CD200	2.97E-01	
CD200R1	4.32E-01	
CD27	1.74E-01	
CD274	8.58E-01	
CD276	2.51E-01	
CD28	4.11E-02	
CD40	4.13E-01	
CD80	7.69E-02	
CD86	5.38E-01	
CEACAM1	6.82E-01	
CTLA4	1.32E-04*	stage III
HAVCR1	1.88E-01	
HAVCR2	8.09E-01	
ICOS	7.83E-02	
IDO1	2.09E-01	
IL2RB	1.96E-01	
LAG3	6.13E-02	
LAIR1	1.71E-02	
PDCD1	6.28E-02	
PDCD1LG2	9.11E-01	
PVR	3.62E-01	
PVRL2	1.22E-01	

TIGIT	1.65E-02	
TNFRSF14	5.99E-01	
TNFRSF18	6.45E-03	
TNFRSF4	3.16E-01	
TNFRSF9	1.00E-02	
TNFSF14	8.19E-02	
TNFSF4	4.20E-01	
TNFSF9	9.04E-03	
VTCN1	4.64E-01	

Supplementary Table 4. Differential expression results of immune checkpoint genes by mutation status (mutated vs. wild-type). Unadjusted P-value shown for each gene and mutation. Asterisk (*) indicates the gene was significantly differentially expressed (FDR <5%) in a transcriptome-wide analysis, with direction of effect noted in "If DE".

ICB Gene	***EGFR* mutation (n=53)**		***STK11* mutation (n=59)**		***KRAS* mutation (n=160)**		***KEAP1* mutation (n=39)**	
	**P-value**	**If DE, increased in:**	**P-value**	**If DE, increased in:**	**P-value**	**If DE, increased in:**	**P-value**	**If DE, increased in:**
BTLA	6.41E-02		4.72E-02		2.04E-01		2.31E-01	
CD200	8.73E-01		1.76E-01		5.48E-01		5.53E-01	
CD200R1	1.72E-01		6.60E-01		4.79E-02		2.67E-01	
CD27	7.20E-01		5.79E-01		6.37E-01		1.33E-01	
CD274	3.94E-01		2.59E-04*	WT	2.64E-02		3.33E-02	
CD276	1.65E-03*	WT	3.30E-01		1.89E-04*	KRAS mut	8.62E-01	
CD28	4.08E-01		1.04E-02		7.38E-01		8.21E-02	
CD40	5.87E-01		4.26E-04*	WT	8.35E-01		3.66E-02	
CD80	4.90E-02		2.04E-04*	WT	2.81E-01		1.03E-02	
CD86	2.40E-01		7.82E-03		6.99E-01		3.08E-02	
CEACAM1	8.57E-03		7.66E-02		2.35E-01		3.65E-02	
CTLA4	2.01E-01		3.01E-01		2.72E-01		1.41E-01	

HAVCR1	4.43E-01		4.75E-03*	STK11 mut	4.06E-02		5.17E-10*	KEAP1 mut
HAVCR2	5.76E-01		8.32E-03		3.38E-01		8.94E-02	
ICOS	8.38E-02		5.46E-02		8.09E-01		1.48E-01	
IDO1	1.57E-01		5.99E-01		9.53E-01		6.76E-01	
IL2RB	8.84E-01		6.35E-01		4.41E-01		6.17E-01	
LAG3	2.42E-01		9.89E-01		7.99E-01		5.42E-01	
LAIR1	4.69E-01		1.59E-02		7.50E-01		5.23E-01	
PDCD1	5.90E-01		6.87E-01		4.51E-01		5.90E-01	
PDCD1LG2	6.70E-01		1.65E-01		6.91E-01		5.19E-02	
PVR	1.06E-02		1.13E-01		1.73E-04*	KRAS mut	2.47E-01	
PVRL2	1.52E-02		2.81E-01		2.02E-05*	KRAS mut	5.38E-01	
TIGIT	4.08E-01		1.53E-01		8.82E-01		2.77E-01	
TNFRSF14	8.68E-01		8.02E-02		1.05E-01		2.29E-01	
TNFRSF18	9.60E-01		1.59E-01		3.70E-01		9.11E-02	
TNFRSF4	1.02E-01		3.50E-01		1.26E-01		6.91E-01	
TNFRSF9	1.53E-01		1.38E-02		9.11E-01		3.74E-02	
TNFSF14	3.95E-01		1.27E-02		5.77E-02		5.86E-04*	KEAP1 mut

TNFSF4	9.62E-01		6.15E-01		7.44E-01		4.57E-02	
TNFSF9	3.40E-01		2.84E-01		2.64E-01		3.27E-02	
VTCN1	1.04E-01		4.59E-01		1.71E-04*	WT	1.67E-01	
